# Prescription Practices of Cannabinoids in Children with Cerebral Palsy Worldwide—A Survey of the Swiss Cerebral Palsy Registry

**DOI:** 10.3390/children10121838

**Published:** 2023-11-23

**Authors:** Sandra Hunziker, Federico Morosoli, Kathrin Zuercher, Anne Tscherter, Sebastian Grunt

**Affiliations:** 1 Institute of Social and Preventive Medicine, University of Bern, Mittelstrasse 43, CH-3012 Bern, Switzerland; 2 Division of Neuropediatrics, Development and Rehabilitation, Children’s University Hospital, Inselspital, University of Bern, Freiburgstr. 15, CH-3010 Bern, Switzerland

**Keywords:** cannabis, cannabinoids, tetrahydrocannabinol, cannabidiol, cerebral palsy, pediatrics

## Abstract

Cannabinoids are prescribed to children with cerebral palsy despite limited evidence. We aimed to assess cannabinoid prescribing practices in children with cerebral palsy, focusing on indications, types of preparations used, and tolerability. Furthermore, we investigated how physicians acquire knowledge about cannabinoid medication. We asked physicians with expertise in the care of children with cerebral palsy about their prescribing practices for cannabinoids. Data were collected through an online survey, which was distributed by email. In addition to the demographic information of participants, we also inquired about the indications for the prescription of cannabinoids, experiences regarding efficacy, and observed side effects of the therapy. Seventy physicians from Europe, North America, and Australia completed the survey. Forty-seven participants were experienced in treating of children with cerebral palsy with cannabinoids. The most common indication was epilepsy (69%), followed by spasticity (64%) and pain (63%). The preparations and doses prescribed varied considerably. Half of the participants evaluated the effect of the cannabinoids as moderate. Twenty-nine physicians reported side effects, most frequently, drowsiness (26%), somnolence (19%), fatigue (13%), and diarrhea (13%). Despite the lack of evidence to date, cannabinoids are used to treat children with cerebral palsy in a wide variety of indications. Randomized controlled trials in this vulnerable patient group are therefore of utmost importance.

## 1. Introduction

The term cerebral palsy (CP) refers to a non-progressive disorder of posture and movement due to a non-progressive malformation or lesion in the brain [1]. A distinction is made between ataxic, dyskinetic, and spastic (unilateral or bilateral) forms of CP [2]. In addition to motor symptoms, children with CP suffer from a variety of comorbidities, including epilepsy, intellectual impairment, behavioral, musculoskeletal, and nutritional issues, sleep problems, and pain [1,3,4,5,6,7,8]. A wide variety of therapeutic approaches and drug treatments have been described for the individual symptom complexes of patients with CP [9]. Medications are often prescribed to treat epilepsy as well as movement disorders [5,10,11,12]. In addition to conventional medications, cannabinoids are increasingly being used for various indications in children. Cannabis (Cannabis sativa) is a plant-based drug composed of more than 100 phytocannabinoids. Of these, the psychoactive substances Δ9-tetrahydrocannabinol (THC) and cannabidiol (CBD) are of particular interest.

CBD has an anti-inflammatory, anti-epileptic, and anxiolytic effect [13] and is used in children primarily to treat epileptic seizures, anxiety disorders, and psychoses [14,15,16,17,18]. Its use in epilepsy has been well studied. Three randomized controlled trials (RCTs) studied the effect of CBD in patients with Lennox–Gastaut syndrome and Dravet syndrome and showed a greater reduction in seizure frequency compared to placebo with good tolerance [15,16,17,18,19]. A case series in children from the UK Medical Cannabis Registry suggests that treatment with cannabinoids (CBD oils and a combination of CBD and THC) may have a beneficial effect on seizure frequency [20].

THC is used in children and adults for a variety of indications [14,21,22]. In adults, the main use is for chronic pain and spasticity in multiple sclerosis [21,22,23]. There is moderate evidence that THC reduces spasticity in adults with multiple sclerosis [22,23,24], but the evidence for the treatment in other indications for adults is weak [22]. In children and adolescents, the use of THC has been described in patients with neurodegenerative diseases, traumatic brain injury, epilepsy [18,19], posttraumatic stress disorder, and Tourette’s syndrome [14], to treat chemotherapy-induced nausea and vomiting [25], and to treat spasticity in the context of pediatric palliative care [26]. To date, with the possible exception of epilepsy, there is little evidence of efficacy in children for these indications [27]. In January 2019, a systematic literature review summarized the literature on the use of cannabinoids for spasticity, with a special focus on children [28]. A conclusion regarding the effect of cannabinoids on childhood spasticity could not be drawn. Shortly afterwards (March 2019), an RCT on the effect of Nabiximols (Sativex^®^) in 72 children with spasticity (mostly with spastic CP) was published. It showed no significant improvement in spasticity 12 weeks after treatment compared to placebo [29].

Despite the limited evidence for cannabinoids in children (with the exception of CBD in Lennox–Gastaut and Dravet syndrome) [30] and the knowledge of drug interactions [31], cannabinoids seem to have a wide range of indications in children with CP. Information on prescribing practices is mostly based on anecdotal reports and case descriptions. A recent survey of Swiss parents showed that THC and CBD are mainly used in children with neurological disorders [32]. It remains unclear in which context and for which indications cannabinoids are prescribed to children with CP and what is the experience of specialists in treating children with CP with cannabinoids. 

Our goal was to determine the prescription indication, formulation, physician perceived effect, and side effects of cannabinoids, as well as the characteristics and acquisition of knowledge about cannabinoids by these physicians. To answer these questions, we developed a questionnaire for physicians in Switzerland and abroad who work in the field of pediatrics, care for patients with cerebral palsy, or are considering prescribing cannabinoids.

## 2. Materials and Methods

### 2.1. Study Design and Data Collection

This retrospective cross-sectional study collected data from physicians treating children (<18 years) with CP. A questionnaire was developed in English and reviewed by the steering board members of the Swiss Cerebral Palsy Registry. The revised questionnaire (Supplementary Table S1) was used for data collection through an online survey on the SurveyMonkey^®^ platform (San Mateo, CA, USA; www.surveymonkey.com (accessed on 1 September 2019)). Access to the questionnaire was given by e-mail. The target participants were physicians in Europe, North America, and Australia with expertise in the care of children with CP. The initial address list (*n* = 102) was developed by the Steering Board of the Swiss Cerebral Palsy Registry in collaboration with the Swiss Academy of Childhood Disability and was a convenience sample of personal contacts. Using chain-sampling referral, addressees were invited to forward the survey to relevant colleagues (*n* unknown). Data collection took place from October 2019 to February 2020. 

This study was designed as a cross-sectional survey using convenience sampling to explore the current cannabinoid prescribing practices and potentially uncover areas of research. Chain-sampling referral was chosen to ensure a meaningful sample size and to reach the target population [33].

The questionnaire was composed of three sections: (I) four questions to assess if the participants fulfilled the inclusion criteria; (II) demographic data such as sex, age, country of residence, medical specialization, work experience, and workplace of the participants; (III) prescribing practices of cannabinoids in children with CP, including years of experience of the participants, indications and formulas used, observed efficacy, and short-/long-term adverse effects.

### 2.2. Inclusion Criteria and Definition of Groups

Participants fulfilled the inclusion criteria and were given access to the complete questionnaire if they answered positively to one or more of the questions of section (I):

- Have you already worked with patients treated with cannabinoid drugs?
- Were any of them of pediatric age (0–18 years)?
- Were any of these children/adolescents diagnosed with cerebral palsy?
- Have you ever experienced a situation in which the prescription of cannabinoids in this type of patient was debated, but for some reason not prescribed?

Physicians who answered negatively to all four questions or who did not complete the questionnaire were excluded from the study. Participants affirming all four questions of section (I) were defined as experienced in treating children with CP with cannabinoids, while participants affirming one to three questions were defined as less experienced. 

### 2.3. Statistical Analysis

We used descriptive statistics to characterize participants according to experience with cannabinoids in children with CP (yes vs. no) and according to region and assessed the differences between groups using chi-square, Fisher’s exact, or Wilcoxon rank-sum tests. All analyses were performed in Stata (version 15.1, College Station, TX, USA).

### 2.4. Ethical Statement 

This study did not require institutional review board approval under Swiss law.

## 3. Results

**Scheme 1:**
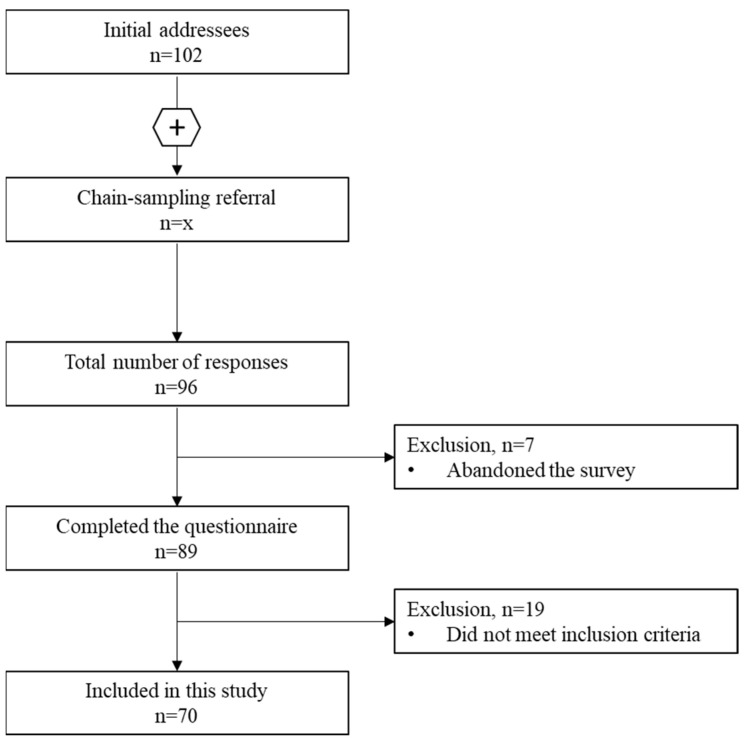
Inclusion flowchart of study participants.

We invited 102 physicians to complete the survey and to forward it to relevant colleagues. A total of 96 physicians responded to the survey. We excluded 7 physicians who did not complete the survey and 19 physicians who did not meet at least one of the four inclusion criteria. Hence, 70 participants were included in the study (Scheme 1). Of these, 23 (33%) belonged to the less experienced group and 47 (67%) were experienced in prescribing of cannabinoids to children with CP.

### 3.1. Study Participants

The median age of participants was 48 years (interquartile range 42–57), and 43 (61%) were female (Table 1). Most were pediatricians (45, 64%), followed by rehabilitation medicine physicians (28, 40%), neurologists (10, 14%), and an anesthetist (1, 1%). Only 12 (17%) participants had less than 5 years’ of work experience in their specialty. The work experience in the treatment of children and adolescents with CP was similar between groups (experienced vs. less experienced, *p* = 0.8). Participants with experience were more likely to work in any hospital (university and/or general hospital) than less experienced participants (42, 89% vs. 14, 61%; *p* = 0.01). Most participants indicated that their main source of knowledge about cannabinoid treatment was individual learning (45, 64%), followed by colleagues (28, 40%), conferences (17, 24%), and as part of their continuing education (15, 21%). Ten (14%) indicated that they had been informed about cannabinoid therapy options by patients and families.

**Figure 1:**
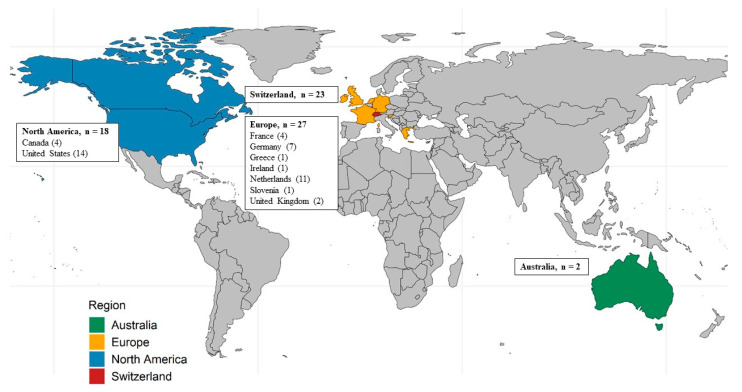
Map showing the country of residence of the participating physicians by region.

Most participants came from Europe (27, 39%), followed by Switzerland (23, 33%), North America (18, 26%), and Australia (2, 3%; Figure 1). Characteristics of the participating physicians by region are provided in Supplementary Table S2.

### 3.2. Indications

The most frequent indication for cannabinoid treatment in children with CP was epilepsy (48, 69%), followed by spasticity (45, 64%), pain (44, 63%), behavioral problems (12, 17%), sleep disturbance (11, 16%), and dystonia (8, 11%; Table 2). Experienced participants were more likely to indicate cannabinoids for epilepsy and spasticity (*p* < 0.05), and a tendency towards pain (*p* = 0.06), compared to the less experienced ones. Cannabinoids were most frequently prescribed as a co-medication (28, 40%) and as a second-line treatment (16, 23%). The most common reasons for not initiating cannabinoids in children with CP were a lack of cost coverage (24, 34%), age of the patient (19, 27%), lack of evidence on effectiveness and side effects (15, 21%), and parents’ wish (13, 19%).

### 3.3. Prescribed Formulas

Experienced participants predominantly prescribed dronabinol solution (Δ9-THC 2.5%; 30%) and cannabis oil (Δ9-THC/CBD = 11/24 mg per g; 17%) among the standardized formulas (Table 3). However, there was a large proportion of participants (12, 26%) who used self-medications (variable contents of Δ9-THC and CBD). Six (13%) participants did not provide specific information in response to this question. Participants with less experience predominantly prescribed cannabis oil ((Δ9-THC/CBD = 11/24 mg per g; 13%), CBD (9%), dronabinol solution (Δ9-THC 2.5%; 9%), and cannabis sativa spray (Δ9-THC/CBD = 2.7/2.5 mg per spray; 9%), but almost half of them (48%) did not provide information.

### 3.4. Perceived Effect of the Treatment and Side Effects

The perceived effect of cannabinoid treatment was reported to be strong or moderate by 32 (69%), weak by 10 (21%), and insignificant by 1 (2%) of the experienced participants (Table 4). Twenty-nine (41%) participants reported short-term side effects, and more experienced participants reported side effects than less experienced ones (25, 53% vs. 4, 17%, *p* = 0.004). The most frequently reported side effects among the experienced participants were drowsiness (12, 26%), somnolence (9, 19%), fatigue (6, 13%), and diarrhea (6, 13%). Less frequently reported side effects are listed in Table 4. No long-term side effects were described.

## 4. Discussion

This international online survey assessed the prescribing practices of cannabinoids in children with CP by their treating physicians. The participating physicians (*n* = 70) acquired their knowledge about cannabinoids mainly outside their medical training. The physicians frequently prescribed differing formulas of cannabinoids for various indications in children with CP. The most common indications were epilepsy, spasticity, and pain, and treatment was initiated as co-medication or second-line treatment. Overall, physicians perceived a moderate efficacy of cannabinoids and no long-term side effects.

The survey included participants form a number of countries. Most participants who prescribed cannabinoids for children with CP worked in clinics, especially in university hospitals, while participants with less experience in prescribing these drugs often worked in rehabilitation centers. Participants indicated that their knowledge of the topic was primarily acquired through individual learning. This highlights the need to promote knowledge transfer and education on the topic. 

In our study, epilepsy was among the three most frequently reported indications for cannabinoids in children with CP. These prescriptions are probably based on the benefit of cannabinoid treatment of specific forms of epilepsy in children [15,16,17,18,19]. However, the verification is likely based on the use of CBD compounds in children with Lennox–Gastaut syndrome or Dravet syndrome. Physicians seem to use cannabinoids to treat epilepsy in young CP patients with associated epilepsy based on these findings. We cannot assess which forms of epilepsy were treated with cannabinoids and whether they were used as the sole substance or as an add-on therapy. Also, it remains unclear whether cannabinoids were used to treat epilepsy or other symptoms.

Spasticity was also among the most often reported indications for cannabinoids in our survey. Cannabinoids are used to treat spasticity in adult CP patients [21,22,23,24], and the treatment of young patients is an ongoing debate [34]. The high prescription rate of cannabinoids for spasticity in children with CP among participants in our study could be derived from the evidence in adults. However, a recent RCT showed no benefit of cannabinoids in the treatment of spasticity in children with CP [29]. We conducted our survey before the publication of this RCT. This recent finding may reduce the prescription rate of cannabinoids for the treatment of spasticity in children with CP in the future.

The third most common indication of cannabinoids in children with CP was pain. This finding was expected, since cannabinoids are used to treat chronic pain of various etiologies [35,36], and chronic pain is a common problem in children with CP, especially in more severe forms [37]. However, the efficacy of cannabinoids to relieve neuropathic pain in children is not supported [14]. There are no studies on the use of cannabinoids for the specific treatment of pain in children with CP. 

An interesting aspect of the survey was the responses regarding dystonia. Several colleagues independently indicated dystonia as the most important indication in the personal comments. To date, there is little information regarding the use of cannabinoids in dystonia. Individual case reports from the adult literature describe positive effects [38]. A pilot study in children with complex movement disorders described improvements in dystonia with the use of cannabinoids [39,40]. 

Our study revealed that a variety of cannabis preparations with widely varying THC and CBD content are used in children with CP. The recurrent use of cannabidiol compounds is probably due to their indication in epilepsy. Unfortunately, from our survey, we cannot determine which substance was used for which indication. In the multiple-choice question addressing the preparations prescribed, we only listed the preparations that are best known to us and most frequently used in Switzerland. However, many colleagues stated that they used other preparations. The reasons for the choice of preparations were not asked. We also do not know which preparations are predominantly available in which countries and the legal background of their purchase. The pharmacologically manufactured Nabiximols (Sativex^®^), which is available as standard in many countries, was rarely used. 

The perceived effect of cannabinoids was reported to be moderate by most experienced physicians. However, the survey does not allow conclusions to be drawn about the effectiveness of cannabinoids for various indications. Half of the experienced physicians described short-term side effects, most commonly, drowsiness and somnolence. No long-term adverse effects were perceived. Nonetheless the survey was not designed to cover the safety spectrum of medicinal cannabinoids. Such a survey is not capable of doing so, nor do we wish to make a definitive statement about safety. Still, our findings on perceived side effects are in line with previous research [41,42]. To answer questions on the efficacy and safety of cannabinoids in children with CP, interventional studies with a multi-centric randomized controlled design or thorough single-case studies are mandatory. Such studies should take patient characteristics into account to allow the prediction of side effects. Based on such trials, evidence for indications could be created to serve as a basis for guideline development.

This study has some limitations. The survey format provides insight into the topic and an impression of whether and how colleagues in western countries treat children with CP with cannabinoids. However, our observations are not representative. We cannot determine the response rate since we used convenience sampling and chain-sampling referral. 

Respondents who completed or dropped out of the survey could have introduced selection bias. For example, colleagues who were concerned about the topic may have been more compelled to complete the survey, while colleagues who did not prescribe or were not supportive of cannabinoids may have abandoned the survey at a higher rate, biasing our findings, especially on prescribing rates. Furthermore, the number of participants per region is too small to draw conclusions by region, and the data do not allow statements to be made about national preferences. When physicians were asked about perceived short- and long-term side effects, no definition was given, which further adds to the subjectiveness of the result. In addition, the physician-perceived effectiveness is affected by respondent memory bias. Furthermore, it is not possible to check the quality of the data, since the survey was conducted anonymously.

Accordingly, the results of our study should be interpreted with caution, should not be generalized, and should not be used to draw conclusions regarding the safety and efficacy of cannabinoids in children with CP.

## 5. Conclusions

This survey shows that cannabinoids are prescribed for a wide range of indications in children with CP in western countries, despite the lack of evidence to support their use in this patient group. Accordingly, it is important that further clinical trials clarify for which indications and in which situations the use of cannabinoids is justified. Regarding epilepsy, it seems important to closely examine the use of cannabidiol, specifically in CP patients. Further studies should also include indications, such as pain, behavioral problems, or dystonia. In addition, future research should assess the side effects in detail with clear definitions, research drug–drug interactions, and assess physician and patient perspectives. All of this will require multi-center RCTs or detailed single-case studies, which could be based, for example, on national or international research platforms and patient registries.

## Figures and Tables

**Table 1:** Characteristics of participating physicians, experienced or less experienced in treating children with cerebral palsy with cannabinoids.

	Total *n* = 70 (%)	Experienced *n* = 47 (%)	Less Experienced *n* = 23 (%)	*p*-Value ^2^
Sex				0.96
Male	25 (36)	17 (36)	8 (35)	
Female	43 (61)	29 (62)	14 (61)	
Unknown	2 (3)	1 (2)	1 (4)	
Age in years (interquartile range)	48 (42–57)	47.5 (41–56)	50 (42–60)	0.92
Specialization ^1^				
Pediatrics	45 (64)	33 (70)	12 (52)	0.14
Physical Medicine and Rehabilitation	28 (40)	18 (38)	10 (44)	0.80
Neurology	10 (14)	8 (17)	2 (9)	0.35
Anesthesia	1 (1)	0	1 (4)	0.33
Subspecialization ^1^				
Yes	53 (76)	39 (83)	14 (61)	0.04
Neuropediatric	28 (40)	19 (40)	9 (39)	0.92
Pediatric rehabilitation medicine	21 (30)	18 (38)	3 (13)	0.05
Developmental pediatrics	8 (11)	5 (11)	3 (13)	1.0
Pediatric palliative care	5 (7)	3 (6)	2 (9)	1.0
Pediatric oncology–hematology	1 (1)	1 (2)	0 (0)	1.0
Neurometabolic	1 (1)	0 (0)	1 (4)	1.0
No	17 (24)	8 (17)	9 (39)	
Work experience in specialty in years				0.81
0–5	12 (17)	8 (17)	4 (17)	
6–10	13 (19)	8 (17)	5 (22)	
11–15	10 (14)	8 (17)	2 (9)	
>15	35 (50)	23 (49)	12 (52)	
Work experience in the treatment of children with Cerebral Palsy in years				0.72
0–5	15 (21)	9 (19)	6 (26)	
6–10	16 (23)	11 (23)	5 (22)	
11–15	18 (26)	14 (30)	4 (17)	
>15	21 (30)	13 (28)	8 (35)	
Workplace ^1^				0.01
Any hospital	56 (80)	42 (89)	14 (61)	
University hospital	23 (33)	20 (43)	3 (13)	
General hospital	27 (39)	18 (38)	9 (39)	
University and general hospital	6 (9)	4 (9)	2 (9)	
Rehabilitation center	12 (17)	4 (9)	8 (35)	
Private/Joined practice	2 (3)	2 (4)	0 (0)	
Knowledge Acquisition ^1^				
Individual learning	45 (64)	33 (70)	12 (52)	0.14
Colleagues	28 (40)	19 (40)	9 (39)	0.92
Conferences	17 (24)	11 (23)	6 (26)	0.81
Part of education	15 (21)	10 (21)	5 (22)	0.97
Parents and families	10 (14)	7 (15)	3 (13)	0.84
Institution	8 (11)	6 (13)	2 (9)	0.62
Companies	2 (3)	1 (2)	1 (4)	0.60
Country of Residence (grouped)				0.004
Switzerland	23 (33)	15 (32)	8 (35)	
Europe excluding Switzerland	27 (39)	13 (28)	14 (61)	
North America	18 (26)	18 (38)	0 (0)	
Australia	2 (3)	1 (2)	1 (4)	

^1^ Multiple responses possible. ^2^ Chi-square, Fisher’s exact, or Wilcoxon rank-sum tests.

**Table 2:** Indications for treatment of children with CP with cannabinoids and reasons to not initiate such treatment.

	Total *n* = 70, (%)	Experienced *n* = 47, (%)	Less Experienced *n* = 23, (%)	*p*-Value ^3^
Indication (overall) ^1,2^				
Epilepsy	48 (69)	37 (79)	11 (48)	0.009
Spasticity	45 (64)	34 (72)	11 (48)	0.04
Pain	44 (63)	33 (70)	11 (48)	0.06
Behavioral problems	12 (17)	9 (19)	3 (13)	0.74
Sleep disturbance	11 (16)	8 (17)	3 (13)	0.67
Dystonia	8 (11)	7 (15)	1 (4)	0.26
None	5 (7)	1 (2)	4 (17)	0.04
Context of prescription				0.01
Co-medication	28 (40)	22 (47)	6 (26)	
Second-line treatment	16 (23)	11 (23)	5 (22)	
Palliative treatment	7 (10)	6 (13)	1 (4)	
First-line treatment	2 (3)	2 (4)	0 (0)	
Parents initiated treatment	2 (3)	2 (4)	0 (0)	
Not applicable	6 (9)	2 (4)	4 (17)	
No response	9 (13)	2 (4)	7 (30)	
Reasons to not initiate cannabinoids ^1^				
Lack of cost coverage	24 (34)	19 (40)	5 (22)	0.12
Age of the patient	19 (27)	15 (32)	4 (17)	0.26
Lack of evidence on effectiveness and side effects	15 (21)	10 (21)	5 (22)	0.97
Parents wish	13 (19)	10 (21)	3 (13)	0.52
Drug interaction	10 (14)	8 (17)	2 (9)	0.48
Other co-morbidities	3 (4)	3 (6)	0 (0)	0.55
None	8 (11)	5 (11)	3 (13)	1.00

^1^ Multiple responses possible. ^2^ Most important and further indications grouped. ^3^ Chi-square, Fisher’s exact, or Wilcoxon rank-sum tests.

**Table 3:** Cannabinoids used for the treatment of children with cerebral palsy.

Total	Total *n* = 70, (%)	Experienced *n* = 47, (%)	Less Experienced *n* = 23, (%)
Preparation			
Dronabinol solution: Δ9-THC 2.5%	16 (23)	14 (30)	2 (9)
Self-medication: Diff. contents of Δ9-THC & CBD	13 (19)	12 (26)	1 (4)
Cannabis oil: Δ9-THC/CBD = 11/24 mg per g	11 (16)	8 (17)	3 (13)
Cannabis tincture: Δ9-THC/CBD = 11/22 mg per g	3 (4)	2 (4)	1 (4)
Cannabis sativa spray (Sativex): Δ9-THC/CBD = 2.7/2.5 mg per spray	4 (6)	2 (4)	2 (9)
CBD 2,5,10%	3 (4)	1 (2)	2 (9)
Epidiolex^®^	3 (4)	2 (4)	1 (4)
No information	17 (24)	6 (13)	11 (48)

CBD, cannabidiol; THC, tetrahydrocannabinol.

**Table 4:** Short- and long-term side effects observed in cerebral palsy children treated with cannabinoids.

Total	Total *n* = 70, (%)	Experienced *n* = 47, (%)	Less Experienced *n* = 23, (%)
Effectiveness (physician reported)			
Insignificant	2 (3)	1 (2)	1 (4)
Weak	12 (17)	10 (21)	2 (9)
Moderate	35 (50)	28 (60)	7 (30)
Strong	6 (9)	4 (9)	2 (9)
No response	15 (21)	4 (9)	11 (48)
Short-term side effects ^1^			
Yes	29 (41)	25 (53)	4 (17)
Drowsiness	14 (20)	12 (26)	2 (9)
Somnolence	10 (14)	9 (19)	1 (4)
Fatigue	6 (9)	6 (13)	0 (0)
Diarrhea	6 (9)	6 (13)	0 (0)
Nausea	5 (7)	4 (9)	1 (4)
Anxiety	4 (6)	4 (9)	0 (0)
Confusion	4 (6)	4 (9)	0 (0)
Disorientation	3 (4)	3 (6)	0 (0)
Euphoria	3 (4)	3 (6)	0 (0)
Dry mouth	2 (3)	2 (4)	0 (0)
Hallucination	2 (3)	2 (4)	0 (0)
Vomiting	2 (3)	2 (4)	0 (0)
Weakness	1 (1)	1 (2)	0 (0)
Asthenia	1 (1)	1 (2)	0 (0)
Depression	1 (1)	1 (2)	0 (0)
Dizziness	1 (1)	1 (2)	0 (0)
Paranoia	1 (1)	1 (2)	0 (0)
Psychosis	1 (1)	1 (2)	0 (0)
Hypotonia	1 (1)	1 (2)	0 (0)
Increased liver enzymes	1 (1)	1 (2)	0 (0)
No	41 (59)	22 (47)	19 (83)
Long-term side effects			
Yes	0 (0)	0 (0)	0 (0)
No	41 (59)	35 (75)	6 (26)

^1^ Multiple responses possible.

## Data Availability

The data presented in this study are available on request from the corresponding author. The data are not publicly available as individual answers to the survey could possibly be assigned due to the small sample.

## Supplementary Materials

The following supporting information can be downloaded at: https://www.mdpi.com/article/10.3390/children10121838/s1, Table S1: Online survey questionnaire; Table S2: Characteristics of the participating physicians by region.
