# Supplementary material for: Prescription Practices of Cannabinoids in Children with Cerebral Palsy Worldwide—A Survey of the Swiss Cerebral Palsy Registry
Source: Children (Basel). 2023 Nov 23;10(12):1838. doi: 10.3390/children10121838 (PMC10742030; doi:10.3390/children10121838)
Supplement: Supplementary file 1 [file children-10-01838-s001.zip › children-2659016-supplementary.pdf]

Table S1. Online survey questionnaire.

Q1	Have you already worked with patients treated with cannabinoid drugs?<br>☐ *Yes*<br>☐ *No*
Q2	Were any of these in a paediatric age (0-18 years)?<br>☐ *Yes*<br>☐ *No*
Q3	Were any of these children/adolescents diagnosed with cerebral palsy?<br>☐ *Yes*<br>☐ *No*
Q4	Did you ever experience a situation in which the prescription of medical cannabinoids in this type of patients was debated, but for some reason not prescribed?<br>☐ *Yes*<br>☐ *No*
Q5	Before we continue with the survey, please inform us how you answered the first 4 questions.<br>*(If at least one of the questions Q1-Q4 was answered affirmatively, access to Q6-Q34 was provided.)*<br>☐ *I answered the first four questions all with no*<br>☐ *I answered at least one of the first four questions with a yes*
Q6	What is your country of birth?<br>____________________________
Q7	In what year were you born? (Enter 4-digit birth year; for example, 1976)<br>____________________________
Q8	What is your gender?<br>☐ *Male*<br>☐ *Female*
Q9	Where did you attain your medical degree (enter city and country)?<br>____________________________
Q10	What is your medical specialization?<br>☐ *Paediatrics*<br>☐ *Neurology*<br>☐ *Neurosurgery*<br>☐ *Child and adolescent psychiatry and psychotherapy*<br>☐ *Anaesthesia*<br>☐ *Medical genetics*<br>☐ *Psychiatry and psychotherapy*<br>☐ *Radiology*<br>☐ *Physical medicine and rehabilitation*

☐ *General internal medicine*

☐ *Orthopaedics*

☐ *Paediatric surgery*

☐ *Other (Specify)*

Q11 How many years of experience do you have in your specialty? (If more than one specialty, please specify the numbers for every single one, in the comment box below.)

☐ *0-5*

☐ *6-10*

☐ *11-15*

☐ *>15*

Q12 Have you also attained a paediatric subspecialty?

☐ *Yes*

☐ *No*

Q13 Please specify your subspecialty.

☐ *Developmental paediatrics*

☐ *Paediatric rehabilitation medicine*

☐ *Paediatric palliative care*

☐ *Neuropediatrics*

☐ *Paediatric oncology-haematology*

☐ *Other (specify)*

Q14 How many years of experience do you have in your subspecialty? (If more than one subspecialty, please specify the numbers for every single one, in the comment box below.)

☐ *0-5*

☐ *6-10*

☐ *11-15*

☐ *>15*

Q15 In which country do you currently work?

____

Q16 What kind of institution do you currently work in?

☐ *Hospital*

☐ *Private practice*

☐ *Joined practice*

☐ *University hospital*

☐ *Other (specify)*

Q17 How were you introduced to medical cannabinoids?

☐ *As part of my education*

☐ *Participation in a congress on the theme*

☐ *Individual learning on the subject*

☐ *Through the advice of other collaborators*

☐ *Through education provided by my institution*

☐ *Through education provided by pharmaceutical companies*

☐ *Other (specify)*

Q18 How many years have you been treating children diagnosed with cerebral palsy?

☐ *0-5*

☐ *6-10*

☐ *11-15*

☐ *16-20*

☐ *>20*

Q19 How many of your patients diagnosed with cerebral palsy are/were treated with medical cannabinoids?

____

Q20 How long have you been working with cannabinoids for therapeutic purposes? (years)

☐ *0-5*

☐ *6-10*

☐ *11-15*

☐ *16-20*

☐ *>20*

Q21 What is, in your experience, the most important indication to start a treatment with cannabinoids in children with cerebral palsy?

☐ *Pain*

☐ *Epilepsy*

☐ *Sleep disturbance*

☐ *Spasticity*

☐ *Behavioural problems*

☐ *None*

☐ *Other (specify)*

Q22 How would you assess, on a scale from 1 to 5, the effectiveness of the therapy in the context of the indication above? (Scale values are specified below.)

☐ *Insignificant*

☐ *Weak*

☐ *Moderate*

☐ *Strong*

☐ *Excellent*

Q23 Would you also consider further indications?

☐ *Pain*

☐ *Spasticity*

☐ *Epilepsy*

☐ *Behavioural problems*

☐ *Sleep disturbance*

☐*No*

☐ *Other (specify)*

Q24 In which context was the therapy mainly started?

☐ *First line treatment*

☐ *Second line treatment*

☐ *Co-medication*

☐ *Palliative treatment*

☐ *Other (specify)*

Q25 Have there been any criteria that prevented/suggested not to undertake this therapy in paediatric patients?

☐ *Age of the patient*

☐ *Parents' wishes*

☐ *Other pathologies (contraindications)*

☐ *Lack of cost coverage by the health insurance*

☐ *Legal or administrative hurdles*

☐ *Other medicines (drug interactions)*

☐ *None*

☐ *Other (specify)*

Q26 Which type of drug has mostly been used? (The list below only lists the preparations offered in Switzerland, if you have prescribed other preparations, please specify below.)

☐ *Cannabis sativa spray (Sativex): viscous extract of Δ9-THC - and CBD, standardized contents: 2.7/2.5 mg per spray*

☐ *Dronabinol solution: synthetically produced Δ9-THC, standardized dronabinol content 2,5%*

☐ *Cannabis tincture: alcoholic drop solution, standardized Δ9-THC and CBD contents: 11/22mg per g*

☐ *Cannabis oil: oily drop solution, standardized Δ9-THC and CBD contents: 11/24mg per g*

☐ *Sativa oil: oily drop solution, standardized Δ9-THC and CBD contents (Sativex equivalent ratio ☐ 2,7/2,5 mg per g)*

☐ *Self-medication: different contents of Δ9-THC, CBD, and other cannabinoids*

☐ *Other (specify)*

Q27 What was the maximum dosage prescribed for the respective preparations? (Please enter how it was prescribed and the dose of THC and/or CDB in mg/kg/day.)

____

Q28 Was there a habituation effect noticed?

☐ *Yes*

☐ *No*

Q29 Was a dose adjustment necessary as a result?

☐ *Yes*

☐ *No*

Q30 Were any important short-term adverse events noticed?

☐ *Anxiety*

☐ *Asthenia*

☐ *Balance*

☐ *Confusion*

☐ *Depression*

☐ *Diarrhoea*

☐ *Disorientation*

☐ *Dizziness*

☐ *Dry mouth*

☐ *Dyspnoea*

☐ *Euphoria*

☐ *Eye disorders*

☐ *Fatigue*

☐ *Hallucination*

☐ *Nausea*

☐ *Paranoia*

☐ *Psychosis*

☐ *Seizures*

☐ *Somnolence*

☐ *Vomiting*

☐ *Weakness*

☐ *None*

☐ *Other (specify)*

Q31 What consequences have they had for the continuation of the therapy?

☐*Irrelevant*

☐ *Drug dose adjustment*

☐ *Therapy stop*

☐ *Other (specify)*

Q32 Were any important long-term adverse events noticed?

☐ *Cardiovascular disease*

☐ *Respiratory disease*

☐ *Cancer*

☐ *Psychotic disorders*

☐ *Suicide or suicidal thoughts*

☐ *None*

☐ *Other (specify)*

Q33 On a scale from 1 to 5, please give us below a personal rating for your experience with the use of these therapies in children with cerebral palsy.

☐ *Bad*

☐ *Acceptable*

☐ *Average*

☐ *Good*

☐ *Outstanding*

Q34 Remarks.

____

Table S2. Characteristics of the participating physicians by region.

	**Total** n=70, (%)	**Switzerland** n=23, (%)	**Europe** excluding Switzerland n=27, (%)	**North America** n=18, (%)	**Australia** n=2, (%)	**p-value** [3,4]
**Sex**						0.07
Male	25 (36)	0 (0)	5 (19)	0 (0)	0 (0)	
Female	43 (61)	11 (48)	20 (74)	10 (56)	2 (100)	
Unknown	2 (3)	12 (52)	2 (7)	8 (44)	0 (0)	
**Age in years (interquartile range)**	48 (42-57)	49 (46-57)	51 (42-61)	41.5 (38-55)	40 (38-42)	
**Workplace**[1]						<0.001
Any hospital	56 (80)	20 (87)	16 (59)	17 (94)	2 (100)	
University hospital	23 (33)	11 (48)	5 (19)	7 (39)	0 (0)	
General hospital	27 (39)	9 (39)	10 (37)	6 (33)	2 (100)	
University and general hospital	6 (9)	1 (4)	1 (4)	4 (22)	0 (0)	
Rehabilitation centre	12 (17)	0 (0)	11 (41)	1 (6)	0 (0)	
Private/Joined practice	2 (3)	2 (9)	0 (0)	0 (0)	0 (0)	
**Work experience with cannabinoids in years**						0.80
0-5	64 (91)	21 (91)	25 (93)	16 (89)	0 (0)	
6-10	5 (7)	2 (9)	2 (7)	1 (6)	2 (100)	
11-15	1 (1)	0 (0)	0 (0)	1 (6)	0 (0)	
**Indication (overall)**[1,2]						
Epilepsy	48 (69)	17 (74)	13 (48)	16 (89)	2 (100)	0.01
Spasticity	45 (64)	19 (83)	15 (56)	10 (56)	1 (50)	0.08
Pain	44 (63)	19 (83)	14 (52)	10 (56)	1 (50)	0.06
Behavioural problems	12 (17)	5 (22)	5 (19)	1 (6)	1 (50)	0.41
Sleep disturbance	11 (16)	5 (22)	3 (11)	2 (11)	1 (50)	0.57
Dystonia	8 (11)	2 (9)	4 (15)	2 (11)	0 (0)	0.90
None	5 (7)	1 (4)	4 (15)	0 (0)	0 (0)	0.21
**Context of prescription**						0.06

Co-medication	28 (40)	10 (44)	7 (26)	10 (56)	1 (50)	
Second line treatment	16 (23)	6 (26)	4 (15)	4 (22)	0 (0)	
Palliative treatment	7 (10)	5 (22)	2 (8)	0 (0)	0 (0)	
First line treatment	2 (3)	0 (0)	1 (4)	1 (6)	0 (0)	
Parents initiated treatment	2 (3)	0 (0)	2 (7)	0 (0)	0 (0)	
Not applicable	6 (9)	1 (4)	4 (15)	1 (6)	0 (0)	
No response	9 (13)	1 (4)	7 (26)	1 (6)	0 (0)	
**Reasons for not initiating cannabinoids[1]**						
Lack of cost coverage	24 (34)	5 (22)	10 (37)	9 (50)	0 (0)	0.18
Age of the patient	19 (27)	8 (35)	4 (15)	7 (39)	0 (0)	0.12
Lack of evidence on effectiveness and side effects	15 (21)	4 (17)	8 (30)	3 (17)	0 (0)	0.57
Parents wish	13 (19)	4 (17)	4 (15)	5 (28)	0 (0)	0.59
Drug interaction	10 (14)	1 (4)	4 (15)	5 (28)	0 (0)	0.13
Other co-morbidities	3 (4)	0 (0)	1 (4)	2 (11)	0 (0)	0.26
None	8 (11)	6 (26)	1 (4)	1 (6)	0 (0)	0.43

[1]Multiple responses possible

[2]Most important and further indications grouped

[3]Excluding Australia due to low sample size.

[4]Chi-square, Fisher's exact, or Wilcoxon rank-sum tests
